# Dopaminergic encoding of future defensive actions in the mouse nucleus accumbens

**DOI:** 10.1093/pnasnexus/pgaf128

**Published:** 2025-04-29

**Authors:** Austen A Fisher, L Sofia Gonzalez, Zoe R Cappel, Kassidy E Grover, Ronald R Waclaw, J Elliott Robinson

**Affiliations:** Division of Experimental Hematology and Cancer Biology, Department of Pediatrics, Cincinnati Children's Hospital Medical Center, Cincinnati, OH 45229, USA; Department of Pediatrics, University of Cincinnati College of Medicine, Cincinnati, OH 45229, USA; Division of Experimental Hematology and Cancer Biology, Department of Pediatrics, Cincinnati Children's Hospital Medical Center, Cincinnati, OH 45229, USA; Department of Pediatrics, University of Cincinnati College of Medicine, Cincinnati, OH 45229, USA; Neuroscience Graduate Program, University of Cincinnati College of Medicine, Cincinnati, OH 45267, USA; Division of Experimental Hematology and Cancer Biology, Department of Pediatrics, Cincinnati Children's Hospital Medical Center, Cincinnati, OH 45229, USA; Department of Pediatrics, University of Cincinnati College of Medicine, Cincinnati, OH 45229, USA; Neuroscience Graduate Program, University of Cincinnati College of Medicine, Cincinnati, OH 45267, USA; Division of Experimental Hematology and Cancer Biology, Department of Pediatrics, Cincinnati Children's Hospital Medical Center, Cincinnati, OH 45229, USA; Department of Pediatrics, University of Cincinnati College of Medicine, Cincinnati, OH 45229, USA; Neuroscience Graduate Program, University of Cincinnati College of Medicine, Cincinnati, OH 45267, USA; Division of Experimental Hematology and Cancer Biology, Department of Pediatrics, Cincinnati Children's Hospital Medical Center, Cincinnati, OH 45229, USA; Department of Pediatrics, University of Cincinnati College of Medicine, Cincinnati, OH 45229, USA; Division of Experimental Hematology and Cancer Biology, Department of Pediatrics, Cincinnati Children's Hospital Medical Center, Cincinnati, OH 45229, USA; Department of Pediatrics, University of Cincinnati College of Medicine, Cincinnati, OH 45229, USA

**Keywords:** dopamine, nucleus accumbens, visual processing, motivation, fiber photometry

## Abstract

Dopamine release in the nucleus accumbens (NAc) plays a critical role in the motivation to perform actions that promote survival. However, the NAc dopamine response to innately threatening visual stimuli, such as predators descending from above, and the innate behaviors they promote has not been fully characterized. Using the genetically encoded sensor dLight1, we investigated looming visual threat–evoked dopamine release in the lateral (LNAc) and medial NAc shell (NAcS) regions in freely moving mice during performance of a looming stimulus assay. We found that dopamine release related to visual threat perception in the NAcS, but not in the LNAc, predicts the timing and vigor of a future defensive action, yet dopamine released during the performance of the action itself does not. Optogenetic inhibition of dopaminergic terminals in the NAcS at visual stimulus onset prevented escape, confirming a role for ventral striatal dopamine in promoting threat-related behaviors.

**Significance Statement**

Dopamine release in the brain plays a critical role in the motivation to perform actions that promote survival. However, the way in which dopamine systems respond to innately threatening visual stimuli, such as predators descending from above, is not fully understood. In these studies, we show that dopamine released in the brains of mice when they detect a looming aerial threat strongly predicts the timing and vigor of a future defensive behavior, like escape to a shelter. These findings advance our knowledge about how dopamine systems respond to salient visual stimuli that require immediate action to avoid predation.

## Introduction

Dopaminergic projections from the midbrain ventral tegmental area (VTA) to the nucleus accumbens (NAc) in the ventral striatum play an important role in reward processing and motivational control (1–4). While previous research has characterized dopamine responses to sensory stimuli in the context of prediction error detection (5–9), associative learning (10–12), and salience attribution (13–15), less is known about how dopamine systems encode information about innately aversive visual stimuli and the defensive behaviors they promote. In many species, rapidly approaching objects elicit automatic avoidance responses (16–19), such as looming visual threats that mimic predator approach from above and cause rodents to rapidly escape to safety. While many stressors, including looming stimuli (20), increase extracellular dopamine in the NAc (21), the importance of dopaminergic transmission in visually evoked innate defensive behaviors is unclear (22). To address this knowledge gap, we used the genetically encoded sensor dLight1 (23) and fiber photometry to monitor dopamine release in the NAc lateral (LNAc) or medial shell (NAcS) regions in mice while recording mouse behavior during a looming stimulus assay. In this behavioral test, mice are presented with a train of expanding black disks on an overhead display that simulate aerial predator approach from above, which triggers rapid flight to shelter (18). By continuously recording mouse position before and after looming disk presentation, we could determine whether LNAc or NAcS dopamine release correlated with different aspects of defensive behavior, such as escape latency or velocity. Because dopaminergic neurons projecting to the LNAc and NAcS differ in their upstream connectivity and response to aversive stimuli (8, 24), we hypothesized that threat-related dopamine release would differ between the two sites. These studies shed new light on the role of ventral striatal dopamine in the innate defensive response to visual threats.

## Results

**Fig. 1.**
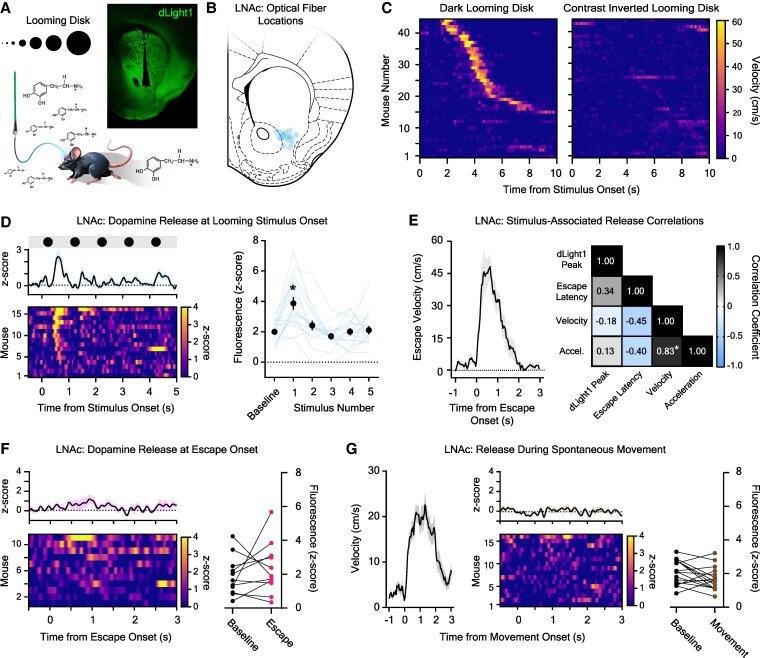
Dopamine responses to visual threats in the LNAc. A) Dopamine release in the mouse NAc was recorded with fiber photometry during a looming stimulus assay. Inset: Representative confocal image with a photometry fiber track and dLight1 expression. B) Photometry fiber tip locations in the LNAc. C) Velocity heat maps showing individual behavioral responses to looming disks (left) and contrast-inverted control disks (right) in all mice tested (*n* = 44 across three groups). D) Left: average (top, *±*SEM) and individual (bottom) LNAc dLight1 responses to black looming disks. Right: the stimulus-associated dLight1 response was dependent upon the stimulus number (*n*_dLight1_ = 16; one-way repeated measures ANOVA with Bonferroni correction; *F*_5,75_ = 8.86, *P* = 0.0004). A significant dopamine transient was observed at the onset of the first disk compared with baseline (*P < 0.05). E) Left: average mouse velocity during escape in mice that fled to the shelter (11 of 16) *±* SEM. Right: Pearson correlation coefficients between the LNAc dLight1 response to the first dark looming disk and escape latency, acceleration, or peak velocity (*P < 0.05). F) Left: average (top, *±*SEM) and individual (bottom) LNAc dLight1 responses at escape onset. Right: no escape-associated change in dLight1 fluorescence was observed relative to baseline (*n* = 11; paired t test; *t*_10_ = 0.85, *P* = 0.42). G) Left: average mouse velocity during a vigorous spontaneous movement bout *±* SEM. Middle: average (top, *±*SEM) and individual (bottom) LNAc dLight1 responses at movement onset. Right: no movement-associated change in dLight1 fluorescence was observed relative to baseline (*n* = 16; paired t test; *t*_15_ = 1.92, *P* = 0.07).

To measure dopamine release in vivo, we performed stereotaxic surgeries to express the dopamine sensor dLight1 in the LNAc or NAcS of C57Bl/6J mice with an AAV vector (AAV5-Syn-dLight1.2). This allowed us to monitor fluorescent dopamine dynamics via an implanted optical fiber with photometry (Fig. 1A and B) while mouse movement was tracked in a custom apparatus containing an escape shelter and an open “threat zone” underneath a display for looming disk presentation (Fig. S1). As expected, a train of five dark looming disks reliably evoked flight to shelter (Fig. 1C, left; peak velocity: 56.3 ± 3.27 cm/s, acceleration: 363.5 ± 34.8 cm/s^2^, escape latency: 3.82 ± 0.29 s, mean ± SEM; 74.4% escape rate across groups). Because mice arrested exploration briefly when they detected looming disks before fleeing (Video S1), dLight1 transients associated with visual stimulus appearance could be temporally distinguished from those associated with escape movements. The ability to evoke escape was unique to black looming disks, as nonthreatening contrast-inverted disks (light gray disks on a black background) (18) did not cause flight to shelter (Fig. 1C, right). When dopamine release was recorded in the LNAc during threat trials, a significant dopamine transient was only observed at the onset of the first looming disk in the stimulus train (dLight1 peak latency: 0.63 ± 0.05 s from stimulus onset; Fig. 1D). These signaling events were not artifacts, since there were no stimulus-induced changes in fluorescence in mice expressing a fluorophore control vector instead of dLight1 (AAV5-Syn-GFP; Fig. S2). The magnitude of the looming disk-evoked dLight1 transient did not predict the decision to escape, given that it was equally robust in mice that fled to the shelter (2.95 ± 0.45 *z*-score) and those that did not (3.22 ± 0.86 *z*-score; *n*_escape_ = 11, *n*_no escape_ = 5; unpaired t test; *t*_14_ = 0.29, *P* = 0.77).

To determine whether LNAc dopamine release encoded information about visual threat-associated defensive behaviors, we performed correlation analysis between stimulus-evoked dLigtht1 peaks, kinematic measurements, and the timing of escape in the subset of mice that fled to the shelter (11 of 16 mice). In escaping mice, dopamine released at looming disk onset did not correlate with the vigor (peak acceleration and velocity) or latency of escape (Fig. 1E), which occurred 1.0–6.22 s after the stimulus-associated dLight1 peak was detected. Next, we aligned the dLight1 signal to movement onset, which allowed us to identify LNAc dopamine released at the time of escape. However, we did not observe statistically significant LNAc dopamine release during escape across mice (Fig. 1F), and the escape-associated peaks identified did not correlate with kinematic features (Fig. S3A and B). To confirm that LNAc dopamine does not reflect movement initiation or ongoing locomotion, we also analyzed the dLight1 signal during vigorous spontaneous movement bouts (Fig. 1G, left; peak velocity: 28.7 ± 1.78 cm/s, acceleration: 284.7 ± 20.6 cm/s^2^) that occurred during the prestimulus exploration period. We did not detect any significant dopamine release during movement bouts outside of the escape context (Fig. 1G, right). Taken together, our results indicate that LNAc dopamine release does not robustly encode information about the decision to escape, the timing and vigor of the future defensive action, or ongoing motor behavior.

**Fig. 2.**
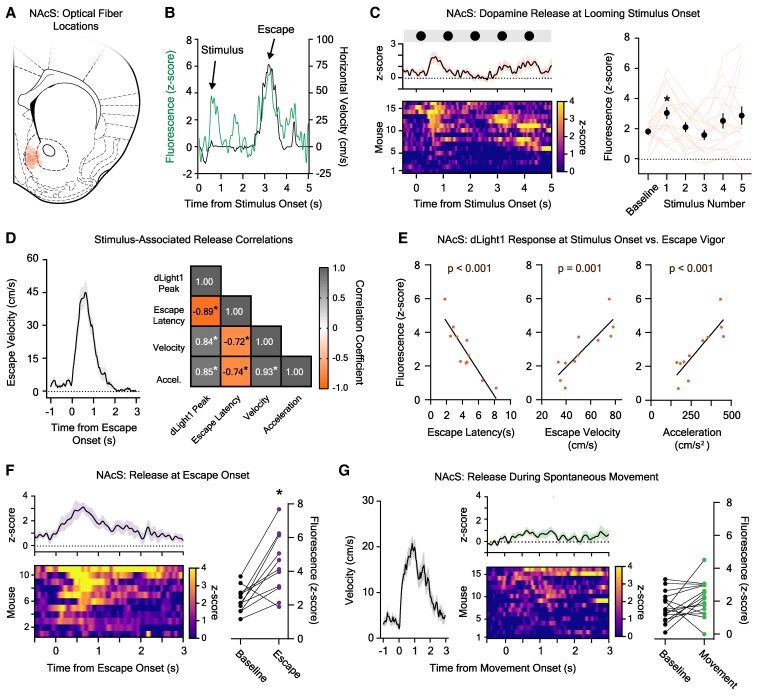
Dopamine responses to visual threats in the NAc medial shell region (NAcS). A) Photometry fiber tip locations in the NAcS. B) Example NAcS dLight1 fluorescence (green) and velocity (black) time traces demonstrating stimulus- and escape-associated dopamine release. C) Left: average (top, *±*SEM) and individual (bottom) NAcS dLight1 responses to a train of five black looming disks. Right: the stimulus-associated dLight1 response was dependent upon the stimulus number (*n* = 16; one-way repeated measures ANOVA with Bonferroni correction; *F*_5,75_ = 3.14, *P*_stimulus number_ = 0.037). A significant dopamine transient was observed at the onset of the first disk when compared with baseline (*P < 0.05). D) Left: average mouse velocity during escape in mice that fled to the shelter (11 of 16) *±* SEM. Right: Pearson correlation coefficients between the NAcS dLight1 response to the first dark looming disk and escape latency, acceleration, or peak velocity (*P < 0.05). E) There were significant correlations between the dLight1 stimulus peak and escape latency (left; *R*^2^ = 0.80, *P* = 0.0002), peak escape velocity (center; *R*^2^ = 0.71, *P* = 0.001), and acceleration (right; *R*^2^ = 0.73, *P* = 0.0008). F) Left: average (top, *±*SEM) and individual (bottom) NAcS dLight1 responses at escape onset. Right A significant dLight1 transient was observed at the onset of escape (*n* = 11; paired t test; *t*_10_ = 4.02, *P* = 0.002) relative to baseline. G) Left: average mouse velocity during a vigorous spontaneous movement bout *±* SEM. Middle: average (top, *±*SEM) and individual (bottom) LNAc dLight1 responses at movement onset. Right: no movement-associated change in dLight1 fluorescence was observed relative to baseline (*n* = 16; paired t test; *t*_15_ = 1.81, *P* = 0.09).

Next, we examined dopamine release in the NAcS during the looming stimulus assay (Fig. 2A and B). A significant dopamine transient was observed shortly after the onset of the first disk but habituated rapidly (Fig. 2C; dLight1 peak latency = 0.71 ± 0.05 s from stimulus onset). Like the LNAc, disk-evoked release did not reflect the decision to escape, given that the peak dopamine response at disk onset was not different between “escapers” (2.95 ± 0.45 *z*-score) and “nonescapers” (2.89 ± 0.77 *z*-score; *n*_escape_ = 11, *n*_no escape_ = 5; unpaired t test; *t*_14_ = 0.070, *P* = 0.67). To investigate the relationship between NAcS dopamine release and defensive behaviors, we performed correlation analysis (Fig. 2D) on stimulus-evoked dLight1 peaks and escape characteristics in mice that fled to the shelter (11 of 16 subjects). We found that the magnitude of the visual stimulus-associated dLight1 transient was strongly correlated with the escape latency, peak velocity, and acceleration during the escape bout, which began 1.2–7.42 s later and did not temporally overlap with the dopamine release event. Specifically, mice with large dLight1 responses at threat onset exhibited shorter latency and higher velocity flight to shelter (Fig. 2E). In contrast, mice with lower magnitude stimulus-evoked dLight1 transients escaped later and with lower intensity. Therefore, NAcS dopamine release at the time of looming disk appearance predicted the timing and vigor of the future defensive action.

To identify dopamine release that occurred at the time of escape, we aligned the dLight1 signal to movement onset in escaping mice (Fig. 2F). We detected a second NAcS dopamine transient (*t* = 4.79 ± 0.57 s after stimulus onset) that occurred shortly after the onset of the escape movement (*t* = 4.29 ± 0.55 s) but before shelter entry (*t* = 5.92 ± 0.65 s) and did not overlap in time with the stimulus-associated dopamine transient (Fig. 2B). Within escaping mice, there was no correlation between the magnitudes of the first, visual stimulus-associated dopamine signal and the second, escape-associated signal (*r* = 0.034, *P* = 0.92), confirming that they were separate dopamine signaling events. Surprisingly, the escape-associated NAcS dLight1 transient (Fig. 2F) did not correlate with any kinematic measurement (Fig. S3C and D) despite occurring contemporaneously. To determine whether NAcS dopamine could encode information about movement initiation or motion state outside the threat context, we analyzed the dLight1 signal during bouts of vigorous prestimulus locomotion (Fig. 2G, left; peak velocity: 32.1 ± 1.30 cm/s, acceleration: 250.3 ± 29.8 cm/s^2^). No significant dopamine release in the NAcS was observed during spontaneous movement prior to threat exposure (Fig. 2G). Thus, NAcS dopamine released during escape is threat-dependent and not indicative of the vigor of the ongoing motor action. This is in stark contrast to dopamine released at the time of sensory perception, which was strongly predictive of the intensity and onset of the future defensive action in mice that fled to shelter.

**Fig. 3.**
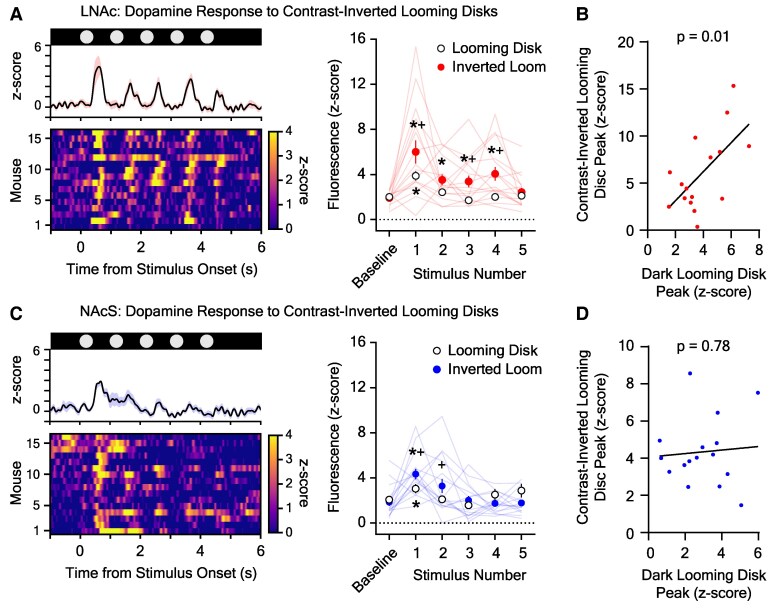
Dopamine responses to nonthreatening contrast-inverted looming disks in the NAc. A) Left: average (top, *±*SEM) and individual (bottom) LNAc dLight1 responses to a train of contrast-inverted looming disks that do not cause flight to shelter. Right: the dLight1 response to expanding disks was dependent upon the stimulus number and stimulus type (*n*_dLight1_ = 16; two-way repeated measures ANOVA with Bonferroni correction; *F*_5,75_ = 2.92, *P*_stimulus type×stimulus number_ = 0.047; *F*_1,15_ = 14.48, *P*_stimulus type_ = 0.0002; *F*_5,75_ = 11.72, *P*_stimulus number_ = 0.0017). Significant LNAc dopamine transients were observed at the onset of the first four contrast-inverted looming disks compared with baseline (*P < 0.05). The dopamine responses to the first, third, and fourth contrast-inverted looming disks were larger than the responses to corresponding black looming disks (^+^*P* < 0.05). B) There was a significant correlation between LNAc dLight1 responses to the first black and contrast-inverted looming disk in the stimulus train (*R*^2^ = 0.39, *P* = 0.01). C) Left: average (top, *±*SEM) and individual (bottom) NAcS dLight1 responses to a train of contrast-inverted looming disks. Right: the dLight1 response to expanding disks was dependent upon the stimulus number and stimulus type (*n*_dLight1_ = 16; two-way repeated measures ANOVA with Bonferroni correction; *F*_5,75_ = 4.21, *P*_stimulus type×stimulus number_ = 0.005; *F*_1,15_ = 0.40, *P*_stimulus type_ = 0.54; *F*_5,75_ = 7.41, *P*_stimulus number_ = 0.0002). Significant NAcS dopamine transients were observed at the onset of the contrast-inverted looming disk compared with baseline (*P < 0.05). The dopamine response to the first and second contrast-inverted looming disks was larger than the response to the corresponding black looming disk (^+^*P* < 0.05). D) There was no correlation between NAcS dLight1 responses to the first black and contrast-inverted looming disk in the stimulus train (*R*^2^ = 0.006, *P* = 0.78).

Dopaminergic neuron firing in the VTA or dopamine release in the NAc can encode the saliency, or relative ethological importance, of a sensory stimulus (13–15). If applicable here, one would predict that more threatening disks would evoke larger dLight1 transients because these stimuli are more relevant to survival (14). To determine whether LNAc and NAcS dopamine tracked threat saliency, we analyzed the dopamine response to nonthreatening, contrast-inverted looming disks (Fig. 3) that have similar edge motion to looming disks but do not evoke escape (18, 20) (Fig. 1C). In both the LNAc (Fig. 3A and B) and the NAcS (Fig. 3C and D), dopamine transients evoked by contrast-inverted disks were larger than those evoked by threatening black disks, indicating that disk-evoked release does not simply encode threat saliency across contexts. This result is consistent with the previous finding that LNAc dopamine can encode information about the rate and magnitude of dark-to-light environmental luminance changes (20), which are larger for light gray contrast-inverted disks that rapidly expand against a black background. When disk responses were compared within mice, we found that the magnitudes of the dopamine transients evoked by dark and contrast-inverted disks were significantly correlated in the LNAc (Fig. 3B; *r* = 0.63, *P* = 0.01) but not in the NAcS (Fig. 3D; *r* = 0.08, *P* = 0.78). While the exact nature of this observation is unknown, it likely reflects observed differences in the ability of NAcS and LNAc dopamine release to encode sensory and/or behavioral features in different threat contexts.

**Fig. 4.**
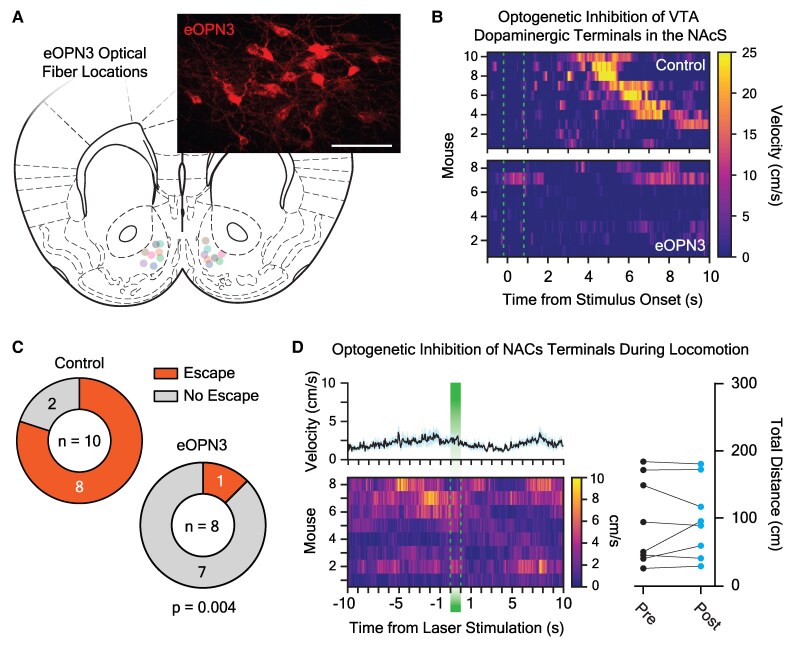
Optogenetic inhibition of NAcS dopaminergic neurotransmission during the looming stimulus assay. A) Optogenetics fiber tip locations in the NAcS of eOPN3-expressing mice. Inset: confocal image showing dopaminergic neurons expressing eOPN3-mScarlett (scale bar = 100 μm). B) Heat maps showing individual behavioral responses to a train of black looming disks when 1 s of 520 nm laser light was delivered to the NAcS 200 ms prior to stimulus onset in mice with or without expression of the inhibitory opsin eOPN3 in dopaminergic neuron terminals. Dashed lines show the onset and termination of the optogenetics lasers. C) Looming disks caused a significantly greater proportion of control mice to escape (8 of 10) than mice expressing eOPN3 in dopaminergic neurons (1 of 8) following NAcS light delivery (520 nm, 10 mW, continuous illumination) at stimulus onset (*n*_control_ = 10, *n*_eOPN3_ = 8; *χ*^2^ goodness-of-fit test; *χ*^2^(1) = 8.10, *P* = 0.004). D) Left: Heat map showing average (top, *±*SEM) and individual (bottom) mouse velocity traces 10 s before and after NAcS light delivery (1 s of 520 nm, 10 mW continuous laser illumination; bar and dashed lines) to eOPN3-expressing mice. Right: there was no difference in the total distance travelled/trial during the 10-s epoch immediately before and after photoinhibition of dopaminergic terminals with eOPN3 in freely moving mice (*n* = 8; paired t test; *t*_7_ = 0.37, *P* = 0.72).

Overall, we found that NAcS but not LNAc dopamine at visual threat onset predicts the timing and vigor of a future defensive action without signaling threat saliency or the decision to escape. To test the causative role of visual stimulus-associated NAcS dopamine in the generation of defensive behavior, we briefly silenced VTA dopaminergic neuron terminals in the NAcS with the inhibitory opsin eOPN3 (25) at the onset of the looming disk train (Fig. 4, Video S2). To facilitate these experiments, we bilaterally injected a Cre-recombinase-dependent eOPN3 vector (AAV5-Syn-SIO-eOPN3-mScarlet) into the VTA of *Dat*-IRES-Cre mice or their Cre-negative littermates, followed by implantation of 300 μm optical fibers above the NAcS for light delivery during behavior (Fig. 4A). In mice expressing eOPN3 in VTA dopaminergic neurons, bilateral delivery of 520 nm laser light (10 mW) to the NAcS for 1 s beginning 200 ms prior to disk onset robustly decreased the probability of escape (12.5% escape rate; Fig. 4B and C). This manipulation did not increase the incidence of freezing as a defensive strategy (0% freezing rate), which was previously observed when dorsal periaqueductal gray area (dPAG) neurons that control escape decisions were silenced (26). In contrast, NAcS light delivery had no effect on controls, which escaped to the shelter at a high rate as expected (80.0% escape rate; Fig. 4B and C). The observed results were not caused by nonspecific motor impairment, as the same 1-s optogenetic manipulation (10 mW, delivered once per minute over 5 min) had no effect on spontaneous locomotion in an open field in opsin-expressing mice (Fig. 4D). Therefore, stimulus-evoked NAcS dopamine release is a crucial signal that controls, in part, the motivated behavioral response to looming visual threats.

## Discussion

By performing dLight1 recordings while animals were tracked during performance of a looming stimulus assay, we identified key differences in the ability of dopamine release in the LNAc and NAcS to encode information about the innate defensive response to visual threats. Across all mice and striatal subregions, the amount of dopamine released at the onset of the sensory stimulus did not predict whether a mouse would escape or reflect the saliency of an expanding disk. However, NAcS dopamine release at the time of visual perception in escaping mice was highly predictive of the timing and vigor of the future defensive action. When this stimulus-evoked release was attenuated by brief optogenetic inhibition of VTA dopaminergic neuron terminals in the NAcS, escape was robustly decreased. Therefore, we hypothesize that NAcS dopamine controls the expression of innate defensive behavior by increasing motivational drive (27–30) rather than influencing the decision to escape, which occurs on a faster timescale by superior colliculus (SC) inputs to the dPAG via a well-validated synaptic threshold mechanism (26). Previous studies have found evidence that VTA neurons act downstream of threat detection centers to modulate behavioral responses to looming disks, as optogenetic inhibition or ablation of VTA glutamatergic (31) or GABAergic (22) (but see (31) for conflicting results) projections attenuated disk-evoked escape. Given our findings, it is surprising that photoinhibition of VTA dopaminergic neuron cell bodies during a looming assay primarily affected poststimulus hiding duration in a previous study (22); however, the ability of dopamine to influence defensive behavior is likely terminal field-dependent and could involve regionally controlled release that is independent of cell body firing (32).

In both the NAcS and LNAc, dopamine release was only evoked by the first stimulus in the train of looming disks. While the neurobiological cause of this rapid habituation is unknown, the literature provides important clues. Several studies report that the dopaminergic response to light involves the SC (reviewed by (33)), which receives direct synaptic input from retinal ganglion cells in its superficial layers (34) and innervates both the substantia nigra pars compacta (SNc) and VTA via deep-layer glutamatergic outputs (22, 35–37). The SC also coordinates defensive responses to looming disks (26, 38), and optogenetic stimulation of the SC during dLight1 recordings evokes spontaneous escape and dopamine release in the LNAc (39). When neural responses to looming disks were measured across SC layers by Lee et al. (40), deep-layer neurons exhibited rapid habituation (within ∼1 s) when the stimulus was repeated. This habituation in deep-layer SC neuron firing was stimulus selective, long-lasting, and attributed to activity-dependent depression of synapses that convey local looming signals to wide-field, threat-detector neurons (40). Thus, rapid habituation of the NAc dopaminergic response to looming disks may involve SC projections to the VTA. However, the involvement of this circuit in the current findings is not clear-cut, as SC neurons primarily form synapses with GABAergic neurons in the VTA (22), and SC control of dopaminergic neurons in rodents mediates orientation of the animal to the visual stimulus (36, 37). Therefore, additional studies will be needed to determine how synaptic inputs from visual processing centers modulate the dopaminergic response to looming visual threats.

We also observed that the dopaminergic response to nonthreatening, contrast-inverted looming disks evoked significantly more dopamine release than threatening looming disks in both the LNAc and NAcS. This difference may reflect the value of the stimulus (5), as threatening disks are aversive and, subsequently, have a lower hedonic value than control disks. It could also represent the occurrence of a sensory prediction error (5, 9), whereby dopaminergic neurons fire to signal that a stimulus is qualitatively different from the one that was expected (6, 7). However, testing these hypotheses with expanding disk stimuli is challenging given that LNAc dopamine release is strongly evoked by stimuli that cause rapid environmental luminance changes, such as when the number of bright pixels on the overhead monitor increases during expansion of a contrast-inverted disk (20). At this time, it is unknown whether NAcS dopamine exhibits luxotonic or irradiance-dependent release, which will need to be explored in depth in the future. This is an important consideration for experiments that evaluate the ability of dopamine to encode threat saliency, as changing disk contrast or expansion speed to alter its threateningness also dynamically affects the irradiance of the overhead display, which could influence the results.

In the looming stimulus assay, dopamine release was only observed during escape in the NAcS, although it did not correlate with the latency or kinematics of the defensive action. Because we did not detect an NAcS dopamine transient during the initiation or maintenance of locomotor behavior outside of disk presentation, it is unlikely that escape-associated release is related to performance of an ongoing motor program. Rather, it may be indicative of motivational vigor (41), a highly dimensional defensive state (42), poststimulus valuation (43), or threat relief (44, 45) that occurs when the mouse approaches the shelter (46). Alternatively, the failure to detect correlations between dopamine release and escape parameters could relate to limitations inherent to our experimental setup. For example, we quantified mouse behavior with contour-based tracking, which does not have the resolution to capture fine behavioral motifs, such as eye motion, changes in limb position, or more granular pose features. Furthermore, dopaminergic neurotransmission was measured in bulk with fiber photometry, which is highly robust but lacks the spatial resolution necessary to detect heterogeneity in dopamine release across small microdomains (47), although this is less of a concern in the NAc where axonal arbors are large (48). Given that dopaminergic neuron responses to environmental stimuli are heterogeneous and vary based on projection target (49), fully elucidating the role of ventral striatal dopamine in the innate defensive responses to visual threats will require continued improvements in the resolution of our behavioral and neural activity readouts.

## Materials and methods

### Experimental animals

Experimental subjects were adult male and female C57Bl/6J mice (the Jackson Laboratory stock no.: 000664) and *Dat*-IRES-Cre mice (the Jackson Laboratory stock no.: 006660). Mice had ad libitum access to food and water, and all experimental procedures were performed during the light phase of the 14 h/10 h light/dark cycle (lights on at 06:00 h, lights off at 20:00 h) in the Cincinnati Children's Hospital Medical Center (CCHMC) vivarium. The mice were housed in same-sex groups of two or three after fiber implantation surgeries. Following the completion of experiments, mice were transcardially perfused with 4% paraformaldehyde in phosphate-buffered saline so that the location of the photometry fiber (shown in Figs. 1B and 2A) or optogenetics fibers (Fig. 4A) could be determined histologically post hoc. Mice were excluded from studies if there was no dynamic photometry signal 8 weeks after surgery or if the optical fiber location was found to be outside the target area. Animal husbandry and experimental procedures involving animal subjects were conducted in compliance with the Guide for the Care and Use of Laboratory Animals of the National Institutes of Health and approved by the Institutional Animal Care and Use Committee (IACUC) and by the Department of Veterinary Services at CCHMC under IACUC protocol 2023-0044.

### Surgical procedures

dLight1 surgical procedures, including viral vector injection and optical fiber implantation, were performed as previously described (20, 39). In brief, C57Bl/6J mice were anesthetized with isoflurane (1–4 and 96% oxygen/CO_2_ mixture) delivered through a nose cone at a rate of 1 L/min. The skull was fixed using a stereotaxic frame (David Kopf Instruments), the scalp was cleaned with chlorhexidine surgical scrub, and an incision was made to expose the skull surface. A craniotomy hole was drilled above the location of the virus injection and photometry fiber implantation. Stereotaxic AAV injections were performed using a blunt or beveled 34- or 35-G microinjection needle within a 10-μL microsyringe (NanoFil, World Precision Instruments) controlled by microsyringe pump with SMARTouch Controller (UMP3T-1, World Precision Instruments). dLight1.2 was expressed in the striatum via stereotaxic injection of 800 nL of a AAV5-hSyn-dLight1.2 vector (1 × 10^13^ viral genome/mL, obtained from Addgene; catalog #111068-AAV5) in different ventral striatal subregions depending on the experiment. Injections in the LNAc used the following coordinates: *A*/*P*: +1.2 mm, *M*/*L*: ±1.7 mm, *D*/*V*: −4.2 mm. NAcS injections used the following coordinates: *A*/*P*: +1.4 mm, *M*/*L*: ±0.65 mm, *D*/*V*: −4.7 mm. In the control group, we injected 800 nL of AAV5-hSyn-EGFP (2 × 10^13^ viral genomes/mL, obtained from Addgene: catalog # 50465-AAV5) in the LNAc. For all AAV injections, viral vectors were injected over 10 min and then allowed to permeate the tissue for an additional 10 min before slowly removing the injection needle. After AAV injections, a 6-mm long, 400 μm outer diameter monofiber-optic cannula with a metal ferrule (MFC_400/430–0.66_6 mm_MF1.25_FLT, Doric Lenses Inc.) was lowered to the same coordinates as the viral injection and affixed to the skull with dental cement. Following surgery, mice were allowed to recover on a heating pad and monitored closely for the next 2 days. Carprofen (5 mg/kg, s.c.) was provided acutely for pain relief and for 2 days after surgery.

Surgical procedures for optogenetic manipulations were performed in male and female *Dat*-IRES-Cre mice (the Jackson Laboratory stock no.: 006660) and littermate Cre-negative controls following the same general protocol described above. After the subjects were anesthetized and the surgical site was prepared, bilateral craniotomy holes were drilled above the VTA (*A*/*P*: −3.3 mm, *M*/*L*: ±0.5 mm). A Cre-inducible eOPN3 vector (500 nL; AAV5-hSyn1-SIO-eOPN3-mScarlet-WPRE; 1.4 × 10^13^ viral genomes/mL, obtained from Addgene; catalog # 125713-AAV5) was injected bilaterally into the VTA using the following coordinates: *A*/*P*: −3.3 mm, *M*/*L*: ±0.5 mm, *D*/*V*: −4.2 mm. Following the injection, 6 mm long, 300 μm fiber-optic cannulas (MFC_300/330-0.22_6mm_MF1.25_FLT, Doric Lenses Inc.) for laser light delivery were bilaterally implanted (angled 20° from vertical) above the NAcS using the following coordinates: *A*/*P*: +1.4 mm, *M*/*L*: ±2.24 mm, *D*/*V*: −4.2 mm. Optogenetic cannulas were affixed to the skull using dental cement, and the mice were allowed to recover from surgery as described above.

### Looming stimulus assay with fiber photometry

Looming stimulus experiments were based on the protocols of Yilmaz and Meister (18) and Evans et al. (26) using a custom-built behavioral apparatus detailed in Gonzalez et al. (20). The open-source experimental control software Bonsai was used for the automation of the looming disk presentation (code available at https://github.com/jelliottrobinson/BonsaiLoomStim). Disk stimuli were displayed on a 15.6-inch liquid crystal display located on one of the ends of the acrylic chamber, 40.5 cm above the acrylic chamber floor. The dimensions of the acrylic chamber were (*L* × *W* × *H*): 61 cm × 20.3 cm × 40.6 cm. This acrylic chamber was placed within a dark enclosure fabricated from polyurethane black foam and modular aluminum rails. The testing apparatus was placed on an IR backlight that matched the dimensions of the acrylic chamber for nonvisible illumination of the chamber. To reduce heat transfer from the light, the acrylic chamber was raised 15 mm above the backlight using plastic pedestals. A black IR-lucent shelter was placed on one end of the acrylic chamber, opposite the monitor displaying the overhead looming disk animations. Mice were placed in the arena and allowed to freely explore for 5 min while video was recorded. After 5 min elapsed, the visual stimulus was triggered when the mouse was detected in the “threat zone.” The threat zone was a 20 cm × 20 cm section of the arena located on the end of the apparatus that was opposite the shelter, and the monitor was mounted directly above this location. The looming stimuli (dark or contrast inverted) were presented in a single train of five consecutive presentations with a 0.5-s ISI between each stimulus on separate experimental days. Disks expanded from 0 to 19.5 cm over 0.25 s and froze at full expansion for 0.25 s, encompassing 27° of visual angle. Video was captured for an additional 3 min after the trigger of the animation. During testing, mice were recorded with a Basler acA2040-120um camera with an Edmunds Optics TECHSPEC 6 mm C Series fixed focal length lens. Mouse position and velocity data were analyzed using Ethovision XT software (Noldus Information Technology) and custom Python code.

During the looming stimulus assay, fluorescent dLight1 signals were monitored using an RZ10x fiber photometry system from Tucker-Davis Technologies. The photometry system employed a 465-nm LED for sensor excitation and a 405-nm LED for isosbestic excitation. Light was filtered and collimated using a six-channel fluorescent MiniCube (FMC6_IE(400-410)_E1(460–490)_F1(500–540)_E2(555–570)_F2(580–680)_S) from Doric Lenses that was coupled to the brain implant via a 1-m fiber-optic patch cable (MFP_400/430/1100–0.57_1_FCM-MF1.25LAF). The emission signal from 405 nm isosbestic excitation was used as a reference signal to account for motion artifacts and photobleaching. A first-order polynomial fit was applied to align the 465 nm signal to the 405 nm signal. Then, the polynomial fitted model was subtracted from the 465 nm channel to calculate Δ*F* values. The Δ*F* time-series trace was *z*-scored within epochs to account for data variability across animals and sessions. The animation trigger and photometry signals were synchronized and time stamped via delivery of transistor-transistor logic (TTL) pulses encoded through the Bonsai software. dLight1 peak data were analyzed using Python. When results were compared with a prestimulus baseline, this value was defined as the amplitude of the largest dLight1 peak that occurred within a 1-s epoch occurring immediately before stimulus delivery, which approximated the amplitude of spontaneous release events and/or fluorescent noise.

### Optogenetic inhibition of dopamine release

Prior to the testing, eOPN3 and Cre-recombinase-negative littermate control mice that did not express the opsin were habituated to tethering several times by connecting both implanted optical fibers to the laser diodes with 1 m fiber-optic patch cables and allowing them to explore the looming stimulus apparatus for ∼10 min without stimulus presentation. On the day of testing, mice were tethered to 520 nm laser diodes (Doric Laser Diode Fiber Light Source LDFLS_520/060_520/060) via 1 m fiber-optic patch cables and placed in the looming stimulus apparatus. A rotary commutator was connected in series between each patch cable and the laser diodes to reduce twisting as the mice turned. Laser stimulation (10 mW, 1 s continuous illumination) occurred when the mouse entered the threat zone, and a train of five looming disks (see above) was triggered 200 ms after laser onset. Video capture and behavioral tracking were controlled by Ethovision (Noldus Informational Technology) using a camera mounted over the arena, as described above. One minute after looming disk presentation, the mice were removed from the arena, untethered, and returned to their home cage. To determine whether the optogenetic manipulation affected spontaneous locomotion, mice were placed in a square open field, and 1 s of 520 nm laser light (10 mW, continuous illumination) was delivered to the NAcS five times with a 1-min interstimulus interval while mouse position was tracked in Ethovision. Distance traveled during the 10 s epoch before and after the light stimulus was calculated for each trial and averaged within mice.

### Statistical analysis

Statistical analysis was performed using Python or GraphPad Prism 9 (GraphPad Software, Inc.). All statistical tests performed on data presented in the manuscript are stated in the figure captions and provided in detail with the corresponding data in Dataset S1. For each experiment, statistical tests were chosen based on the structure of the experiment and the dataset. No outliers were removed during statistical analysis. Parametric tests were used throughout the manuscript. When ANOVA (one- or two-way and/or repeated measures) was performed, multiple comparisons were corrected using the Bonferroni correction.

## Supplementary Material

pgaf128_Supplementary_Data

## Data Availability

All data are included in the manuscript and supporting information (see Dataset S1). Code for the generation of looming disks using Bonsai is available at: https://github.com/jelliottrobinson/BonsaiLoomStim. Python code for photometry data analysis is available: https://github.com/jelliottrobinson/Photometry-Analysis.
