# Supplementary material for: Dopaminergic encoding of future defensive actions in the mouse nucleus accumbens
Source: PNAS Nexus. 2025 Apr 29;4(5):pgaf128. doi: 10.1093/pnasnexus/pgaf128 (PMC12046218; doi:10.1093/pnasnexus/pgaf128)
Supplement: pgaf128_Supplementary_Data [file pgaf128_supplementary_data.zip › PNASNEXUS-PNASNEXUS-2024-01374-TR-s01.docx]

**
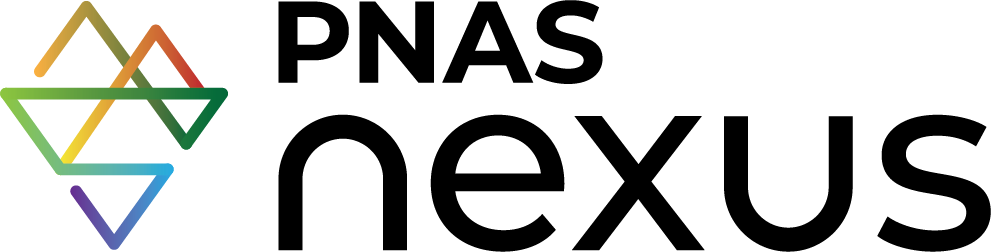
**

**Supplementary Information for**

Dopaminergic encoding of future defensive actions in the mouse nucleus accumbens

Austen A. Fisher, L. Sofia Gonzalez, Zoe R. Cappel, Kassidy E. Grover, Ronald R. Waclaw, and J. Elliott Robinson

Corresponding Author: J. Elliott Robinson

Email: [elliott.robinson@cchmc.org](mailto:elliott.robinson@cchmc.org)

**This PDF file includes:**

Figures S1 to S3

Legends for Video 1 and 2

Legends for Datasets S1

**Other supplementary materials for this manuscript include the following:**

Videos 1 and 2

Datasets S1

**
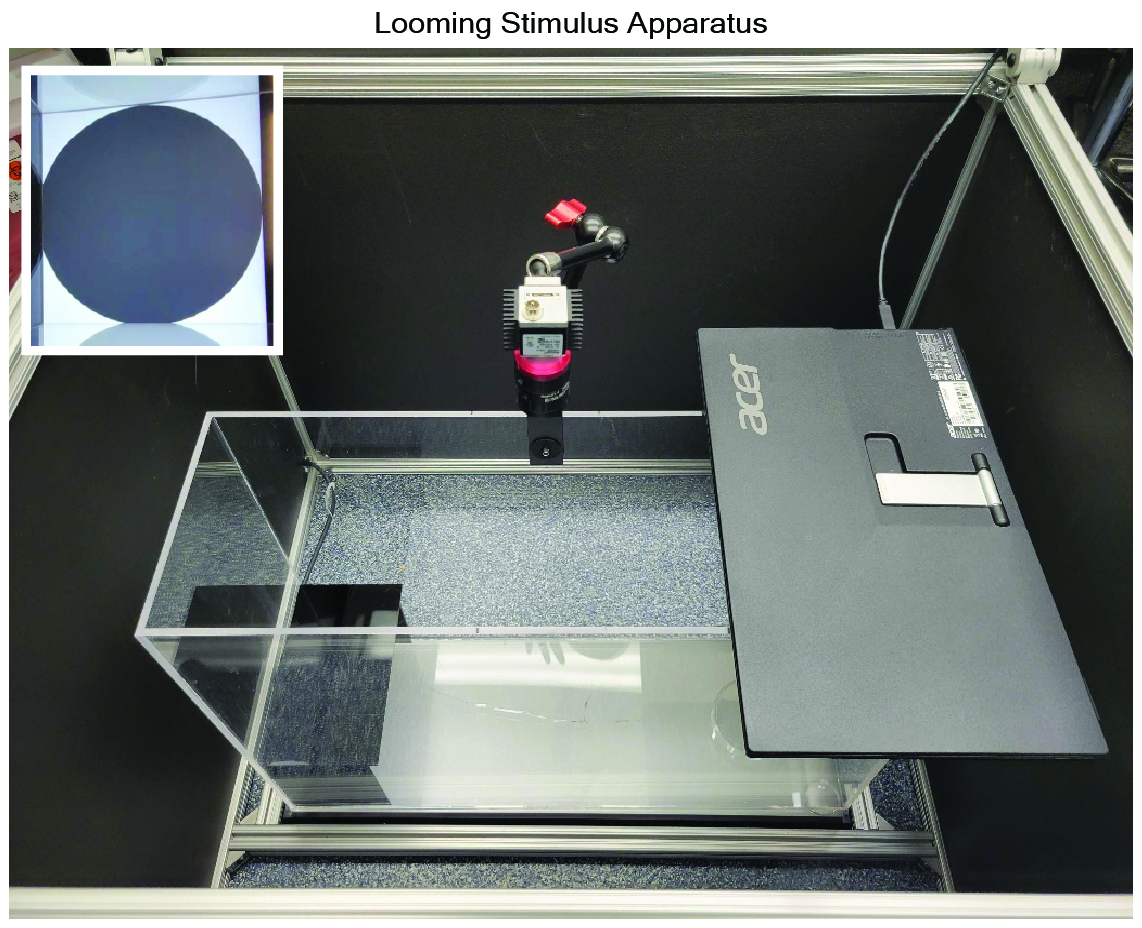
**

**Figure S1. Looming Stimulus Apparatus.** Photograph of the looming stimulus apparatus with an infrared light (IR)-lucent shelter, IR backlight, camera, and display for visual stimulus presentation. The entire apparatus was placed within a dark, sound attenuating enclosure during testing. (*Inset*) Photograph of the monitor used to present expanding looming discs that was above the threat zone in the behavioral apparatus.

**
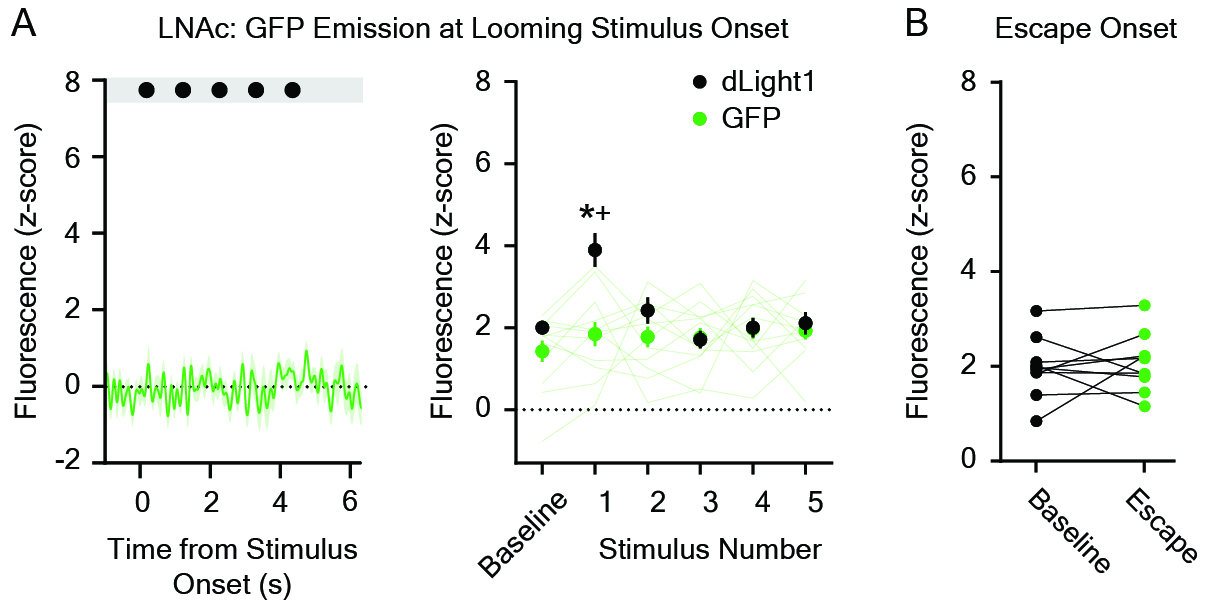
**

**Figure S2. Fluorescence responses to threatening looming discs in GFP control mice. A.** (*Left*) Average (± SEM) LNAc fluorescence emission in response to black looming discs in mice that expressed GFP instead of dLight1. (*Right*) Fluorescence emission in the LNAc in response to threatening looming discs was dependent upon the stimulus number and sensor type (n_dLight1_ = 16, n_GFP_ = 12; 2-way repeated measures ANOVA with Bonferroni correction; F_5,130_ = 7.11, p_sensor type x stimulus number_ < 0.0001; F_1,26_ = 9.58, p_sensor type_ = 0.005; F_5,130_ = 3.11, p_stimulus number_ = 0.03). A significant dLight1 transient was observed at the onset of the first looming disc compared to baseline (* denotes p < 0.05) and GFP control (+ denotes p < 0.05). No significant stimulus-evoked transients were observed in mice expressing GFP instead of dLight1**. B.** There was no significant change in peak fluorescence emission at the onset of escape in mice that expressed GFP instead of dLight1 relative to baseline (n = 10; paired t-test; t_9_ = 0.43, p = 0.70).

**
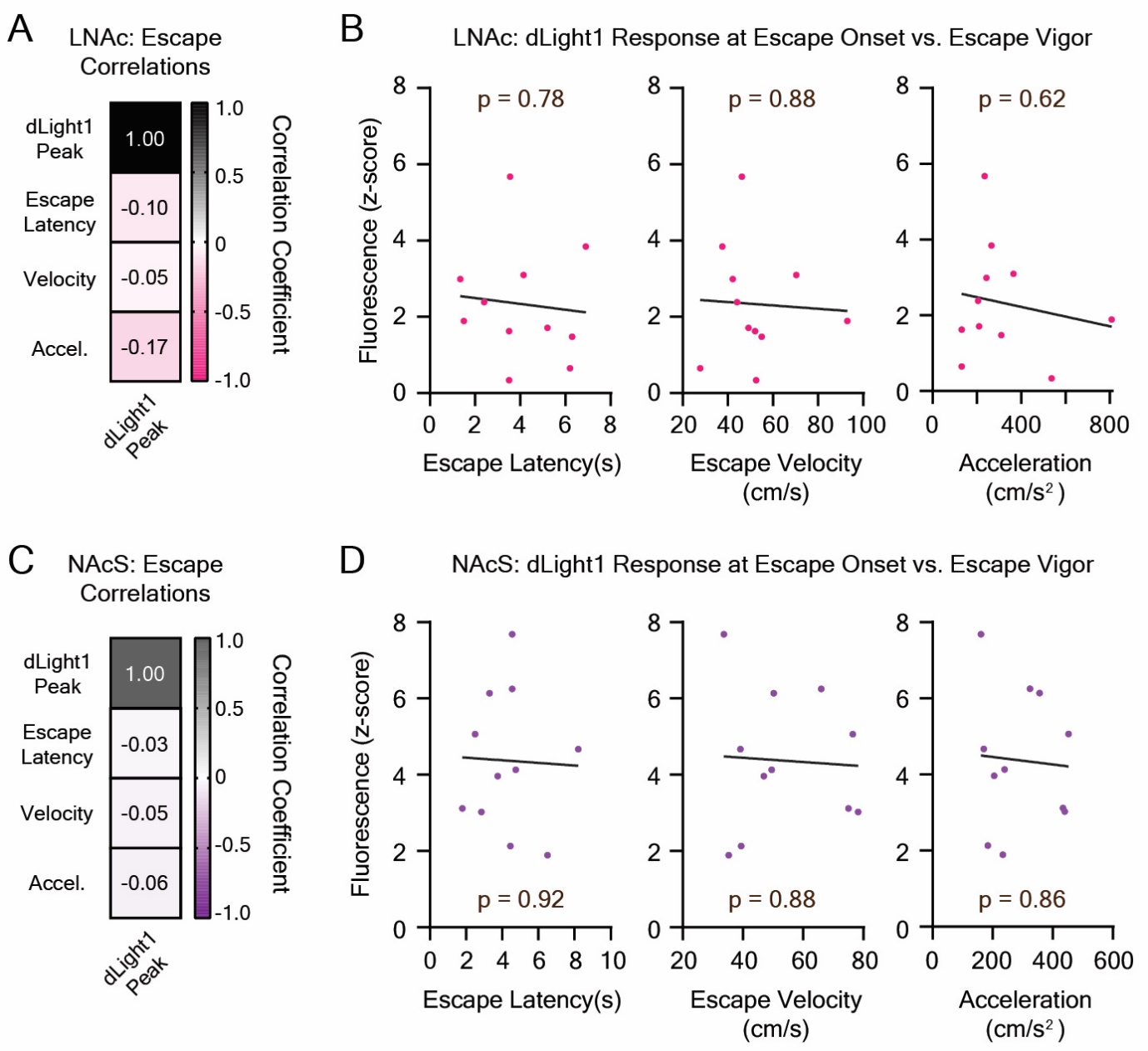
**

**Figure S3. Correlation analysis of escape-associated dopamine transients in the LNAc and NAcS. A.** Pearson correlation coefficients between the peak LNAc dLight1 response at escape onset and escape latency, acceleration, or peak velocity (* denotes p < 0.05). **B.** There were no significant correlations between the escape-associated dLight1 peak and escape latency (*left*; R^2^ = 0.009, p = 0.78), peak escape velocity (*center*; R^2^ = 0.0025, p = 0.88), and peak acceleration (*right*; R^2^ = 0.03, p = 0.62). **C.** Pearson correlation coefficients between the peak NAcS dLight1 response at escape onset and escape latency, acceleration, or peak velocity (* denotes p < 0.05). **D.** There were no significant correlations between the escape-associated dLight1 peak and escape latency (*left*; R^2^ = 0.001, p = 0.92), peak escape velocity (*center*; R^2^ = 0.003, p = 0.88), and peak acceleration (*right*; R^2^ = 0.004, p = 0.86).

**MEDIA CAPTIONS:**

**Video 1.** The behavioral response to the presentation of black looming discs during fiber photometry recordings.

**Video 2.** The behavioral response to looming discs during optogenetic inhibition of VTA dopaminergic neuron terminals in the NAcS with eOPN3.

**DATASET CAPTIONS:**

**Dataset S1.** Source Data and Statistical Testing Results
